# Reviewer Summary for Acute Medicine & Surgery

**DOI:** 10.1002/ams2.927

**Published:** 2024-01-28

*Score Completion Date Range*: Between Jan 1, 2023 and Dec 31, 2023.

The publication of invaluable papers in the Acute Medicine & Surgery depends on the prompt, careful review of submitted manuscripts. We would like to thank the Editorial Board members, and the following experts for reviewing manuscripts from January 1, 2023, to December 31, 2023.

Abe, Ryuzo

Abe, Yoshinobu

Akahoshi, Tomohiko

Amagasa, Shunsuke

Anan, Hideaki

Aokage, Toshiyuki

Aoki, Makoto

Aoki, Yoshihiro

Araki, Takashi

Arimoto, Hideki

Atsumi, Takahiro

Azuma, Kazunari

Banerjee, Pushpal

Boran, Maruf

Doi, Kent

Doi, Tomoaki

Ebihara, Takayuki

Egi, Moritoki

Endo, Tomoyuki

Enomoto, Yuki

Fujii, Tomoko

Fujiogi, Michimasa

Fujisaki, Noritomo

Fujishima, Seitaro

Fujita, Motoki

Fujita, Satoshi

Fujizuka, Kenji

Fukuda, Tatsuma

Fukushima, Hidetada

Funabiki, Tomohiro

Funakoshi, Hiraku

Funazaki, Toshikazu

Furukawa, Makoto

Gando, Satoshi

Goto, Tadahiro

Goto, Yoshikazu

Grensemann, Jörn

Hagiwara, Yusuke

Hanada, Hiroyuki

Hanajima, Tasuku

Hanaki, Nao

Harada, Masahiro

Hatakeyama, Junji

Hattori, Noriyuki

Hayakawa, Koichi

Hayakawa, Mineji

Hayashi, Yasuyuki

Hayashida, Kazuyuki

Hibino, Seikei

Hifumi, Toru

Hirose, Tomoya

Homma, Hiroshi

Homma, Yosuke

Hongo, Takashi

Hosomi, Sanae

Hoyt, David B.

Iba, Toshiaki

Ichiba, Shingo

Ichiba, Toshihisa

Ikeda, Hiroto

Ikezoe, Takayuki

Imai, Yoshiro

Imamoto, Toshiro

Imamura, Hiroshi

Inaba, Mototaka

Inamasu, Joji

Inokuchi, Koichi

Inoue, Akihiko

Inoue, Shigeaki

Inoue, Yoshiaki

Ishida, Kenichiro

Ishihara, Satoshi

Ishikura, Hiroyasu

Isokawa, Shutaro

Iwase, Hiroaki

Iwashita, Yoshiaki

Izawa, Yoshimitsu

Jörres, Achim

Kaita, Yasuhiko

Kajino, Kentaro

Kanda, Jun

Kaneko, Tadashi

Kasaoka, Shunji

Kashiura, Masahiro

Kasugai, Daisuke

Katayama, Yusuke

Kawazoe, Yu

Kikutani, Kazuya

Kinoshita, Takahiro

Kiriu, Nobuaki

Kiyota, Kazuya

Koami, Hiroyuki

Kobata, Hitoshi

Kondo, Hiroshi

Kondo, Yutaka

Kudo, Daisuke

Kurihara, Tomohiro

Kuroda, Yasuhiro

Kurozumi, Taketo

Matsumura, Yosuke

Matsushima, Asako

Matsushima, Hisao

Matsuyama, Shigenari

Matsuyama, Tasuku

Mayumi, Toshihiko

Mitsunaga, Toshiya

Miyamichi, Ryosuke

Miyamoto, Kyohei

Mizushima, Yasuaki

Mohara, Jun

Morimura, Naoto

Morishita, Koji

Moriya, Takashi

Muronoi, Tomohiro

Muroya, Takashi

Nagashima, Futoshi

Naito, Hiromichi

Nakada, Taka‐aki

Nakae, Hajime

Nakagawa, Yuko

Nakamori, Yasushi

Nakamura, Yoshitsugu

Nakao, Atsunori

Ng, Mingwei

Nishimura, Takeshi

Nishimura, Tetsuro

Nishiyama, Kei

Nishiyama, Takashi

Nomura, Osamu

Nosaka, Nobuyuki

Ochiai, Hidenobu

Ohbe, Hiroyuki

Ohshimo, Shinichiro

Okada, Motoi

Okada, Yohei

Omura, Takeshi

Onishi, Shinya

Ono, Hajime

Osawa, Itsuki

Osuga, Keigo

Osuka, Akinori

Otani, Norio

Sakai, Tomohiko

Sakamoto, Ayaka

Sakamoto, Yuichiro

Sakiyama, Yoshio

Sakurai, Atsushi

Sasaki, Junichi

Sasaki, Nobuo

Sasaki, Ryo

Sassoè, Marco

Sato, Nobuhiro

Sato, Tetsuya

Satoh, Kasumi

Satomi, Hidetoshi

Sekine, Kazuhiko

Shibahashi, Keita

Shibusawa, Takayuki

Shime, Nobuaki

Shimizu, K

Shimizu, Keiki

Shinozaki, Koichiro

Shiomi, Naoto

Shiraishi, Atsushi

Suehiro, Eiichi

Takahashi, Nozomi

Takaoka, Makoto

Takeuchi, Ichiro

Takeyama, Naoshi

Tampo, Akihito

Tanaka, Yoshihiro

Tanikawa, Atsushi

Tarui, Takehiko

Tasaki, Osamu

Tsuchiya, Asuka

Tsukahara, Kohei

Tsuruta, Ryosuke

Uchida, Kenichiro

Uehara, Hirofumi

Uemura, Shuji

Ukai, Isao

Umemura, Yutaka

Usui, Ryosuke

Wada, Takeshi

Watanabe, Hiroaki

Yakushiji, Hiromasa

Yamada, Noriaki

Yamakawa, Kazuma

Yamamoto, Ryo

Yamashita, Norio

Yanagawa, Youichi

Yanik, Fazli

Yo, Kikuo

Yokobori, Shoji

Yumoto, Tetsuya

Zhang, Zhongheng

